# Risk Factors for Gambling Disorder: A Systematic Review

**DOI:** 10.1007/s10899-023-10195-1

**Published:** 2023-03-08

**Authors:** Diana Moreira, Andreia Azeredo, Paulo Dias

**Affiliations:** 1 Centro de Solidariedade de Braga/Projecto Homem, Braga, Portugal; 2 Faculty of Philosophy and Social Sciences, Centre for Philosophical and Humanistic Studies, Universidade Católica Portuguesa, Rua de Camões, 60, 4710-362 Braga, Portugal; 3 Faculty of Psychology and Educational Sciences, Laboratory of Neuropsychophysiology, University of Porto, Porto, Portugal; 4 Institute of Psychology and Neuropsychology of Porto – IPNP Health, Porto, Portugal

**Keywords:** Gambling disorder, Pathological gambling, Risk factors, Treatment

## Abstract

Gambling disorder is a common and problematic behavioral disorder associated with depression, substance abuse, domestic violence, bankruptcy, and high suicide rates. In the fifth edition of the Diagnostic and Statistical Manual of Mental Disorders (DSM-5), pathological gambling was renamed “gambling disorder” and moved to the Substance-Related and Addiction Disorders chapter to acknowledge that research suggests that pathological gambling and alcohol and drug addiction are related. Therefore, this paper provides a systematic review of risk factors for gambling disorder. Systematic searches of EBSCO, PubMed, and Web of Science identified 33 records that met study inclusion criteria. A revised study acknowledges as risk factors for developing/maintaining a gambling disorder being a single young male, or married for less than 5 years, living alone, having a poor education, and struggling financially.

For most people, gambling is just an infrequent leisure activity that does not put their lives in danger (Wood & Griffiths, 2015). However, for a small rate of the world population, approximately between 0.12 and 5.8% (Calado & Griffiths, 2016), pathological gambling (PG) is a behavioral disorder. This disorder is defined as an inability to control gambling behavior itself (American Psychiatric Association [APA], 2013), leading to serious health consequences, and financial and legal problems, and representing a risk factor for aggressive behavior (Black, 2022). In the fifth edition of the Diagnostic and Statistical Manual of Mental Disorders (DSM-5), PG was renamed Gambling Disorder and moved to the Substance-Related and Addiction Disorders chapter to acknowledge that PG is associated with alcohol and drug addiction (Black & Grant, 2014).

Custer (1985) describes PG as a multistage disease with different stages of gain, loss, and distress, while the DSM-5 (APA, 2013) describes PG as chronic and progressive. Recent work has shown that PG’s progression is more nuanced and, for most, has its ups and downs. Most players gradually shifted to lower levels of gaming activity, and most experienced spontaneous periods of remission. Research also shows that people who gamble recreationally (or do not gamble at all) are less likely to develop more rigorous levels of gambling activity. Still, some at-risk individuals may experience stressors that push them toward a gambling addiction (Black et al., 2017; LaPlante et al., 2008).

Despite the social and economic toll, there is very little data on predictors of PG progression. Follow-up studies are often small, underpowered, and consist primarily of treatment samples. For example, Hodgins and Peden (2005) re-interviewed 40 PG patients after an average of 40 months. Most tried to stop or reduce gambling, but more than 80% remained problem gamblers. The presence of emotional or substance use disorders was associated with poorer outcomes. Goudriaan et al. (2008) compared 24 PG patients who attended a treatment center with 22 who did not and found that relapsed patients performed worse on disinhibition and decision-making measures. Furthermore, Oei and Gordon (2008) assessed 75 Australian Gamblers Anonymous attendees to assess psychosocial predictors of abstinence and relapse. Those achieving abstinence were more involved in Gamblers Anonymous and reported better social support. More recently, research has investigated the course of gambling disorder in a sample of the general population. In the Quinte study of gambling and problem gambling, Williams et al. (2015) followed 4,121 randomly selected adults for 5 years to assess problematic behavior. They found that being a current problem gambler was the best predictor of future problem gambling. Experiencing “big wins” was also a strong predictor, as was greater gambling intensity.

Several studies have shown a high prevalence of personality disorder (PD) among those with PG, many of which focusing on the association between antisocial personality disorder and gambling (Pietrzak & Petry, 2005; Slutske et al., 2001). On the other hand, Steel and Blaszczynski (1998) observed that almost 53% of pathological gamblers have non-antisocial personality disorder. Other research papers have looked at the co-morbidity of PG with other PD. A recent meta-analysis highlighted that almost half of pathological gamblers show diagnostic criteria for a personality disorder. The majority of these were Cluster B disorders, such as borderline personality disorder, histrionic personality disorder, and narcissistic personality disorder. Other studies looked at comorbidity between PG and disorders from other clusters. There is a consistent comorbidity between PG and paranoid and schizoid personality disorders in Cluster A and with avoidant and obsessive–compulsive personality disorder in Cluster C.

Furthermore, several studies have focused on the overlap between gambling and substance use and have consistently observed significant positive associations between gambling, problem gambling, and alcohol use (Bhullar et al., 2012; Engwall et al., 2004; Goudriaan et al., 2009; Huang et al., 2011; LaBrie et al., 2003; Martens et al., 2009; Martin et al., 2014; Stuhldreher et al., 2007; Vitaro et al., 2001). Gambling is also significantly and positively associated with marijuana and other drug use (Engwall et al., 2004; Goudriaan et al., 2009; Huang et al., 2011; LaBrie et al., 2003; Lynch et al., 2004; Stuhldreher et al., 2007).

The concept of risk implies the concept of hazard and is associated with a high probability of adverse outcomes (Lupton, 1999). That is, risk exposes people to danger and potentially harmful consequences (Werner, 1993). However, risk varies throughout life: it varies according to life circumstances and varies from individual to individual (Cowan et al., 1996). Based on a literature review, Ciarrocchi (2001) described the following risk factors: age, gender, and family background. Pathological gamblers frequently gambled from an early age, suggesting that youth is a risk factor for problem gambling. Also, they are usually male and have relatives who are pathological gamblers (e.g., Cavalera et al., 2018). Regarding family background, some studies have found close relatives with gambling problems, especially parents, to be risk factors for gambling disorder (e.g., Vachon et al, 2004). Kessler et al. (2008) describe several risk factors for gambling disorder: male sex, low educational and socioeconomic levels, and unemployment. After a literature review, Johansson et al., (2009a, 2009b) found that the following groups of risk factors were most frequently reported: (1) demographic variables (under 29; male); (2) cognitive distortions (misperception, illusion of control); (3) sensory characteristics (e.g., (4) reinforcement programs (e.g., operant conditioning); (5) delinquency (e.g., illegal behavior). Regarding older adults, Subramaniam et al. (2015) conducted a study of gamblers aged 60 or older and found that pathological gamblers were more likely to be single or divorced/separated and gambled to improve their emotional state compared to a control group and to compensate for their inability to perform activities of which they were previously capable.

Additionally, the coronavirus disease (COVID-19 pandemic) forced governments to adopt measures such as staying at home and practicing social distancing (Mazza et al., 2020). More adverse measures were also implemented, such as general or regional lockdowns. These stringent measures, associated with reduced social support, economic crises and unemployment, fear of the disease, increased time with the partner and reduced availability of health services, can significantly contribute to the increase of stress in an already strenuous relationship, precipitating or exacerbating gambling problems (Economou et al., 2019; Jiménez-Murcia et al., 2014; Olason et al., 2017). In fact, historically, in economic crises, when people experienced stress due to, for example, isolation, gambling activity per se increased, and so did gambling problems (Economou et al., 2019; Jiménez-Murcia et al., 2014; Olason et al., 2017), but recent studies on potential changes in gambling activity during the COVID-19 pandemic have reported different changes in behavior (Brodeur et al., 2021). One possible explanation might be the restrictions in place in the field of study, along with differences in study populations. Auer et al. (2020) and Lindner et al., (2020) found a substantial decrease in overall gambling activity, especially in gambling, where there were far fewer betting opportunities because of cancelled or postponed sports events such as football leagues.

Many studies have been dedicated to studying risk factors for the development/maintenance of gambling disorder. However, no study has systematically reviewed them to compile them. Therefore, this systematic review aims to explore what are the risk factors for the development/maintenance of gambling disorder. Particularly important if you can see a difference in the pattern between the pre-pandemic and the pandemic crisis.

## Method

### Search Strategy

Studies were identified through search on EBSCO, PubMed, and Web of Science. The reference lists of the selected studies were also reviewed to identify other relevant studies (manual searching). The search equation in **EBSCO** was:

TI (gambling).

 AND TI (“contributing factor*”

 OR predictor*

 OR caus*

 OR vulnerabilit*

 OR outcome*

 OR chang*

 OR barrier*

 OR risk

 OR seek*

 OR treatment*).

In **Pubmed:**

(gambling[Title]).

 AND (“contributing factor*”[Title].

 OR predictor*[Title]

 OR caus*[Title]

 OR vulnerabilit*[Title]

 OR outcome*[Title]

 OR chang*[Title]

 OR barrier*[Title]

 OR risk[Title]

 OR seek*[Title]

 OR treatment*[Title]).

And in **Web of Science:**

(TI = (gambling)).

 AND TI=(“contributing factor*”

 OR predictor*

 OR caus*

 OR vulnerabilit*

 OR outcome*

 OR chang*

 OR barrier*

 OR risk

 OR seek*

 OR treatment*).

The search was limited from the year 2016 and linguistic factors (Portuguese, English, Spanish, or French).

### Study Selection

We had four inclusion criteria and built four corresponding exclusion criteria in response. We wanted population over 18 years old, so we excluded children and teenagers. We wanted only empirical studies, so we excluded all case studies, book chapters, theoretical essays, and systematic reviews (with or without meta-analyses). We only wanted studies involving problem or pathological gambling, so we excluded studies that did not include either of those two. Also, we wanted studies involving risk factors associated with gambling problems, so we excluded studies that did not include it. And we only wanted studies published in the last 6 years (since 2016), so we excluded the others.

The studies were selected by two independent reviewers (DM and AA), based on their titles and abstracts, according to recommendations of PRISMA guidelines (Moher et al., 2009).

The agreement index in the study selection process was assessed with Cohen’s Kappa and revealed almost perfect agreement, *K* = 0.98, *p* < 0.001 (Landis & Koch, 1977). The disagreements among reviewers were discussed and resolved by consensus.

### Identification and Screening

**Fig. 1:**
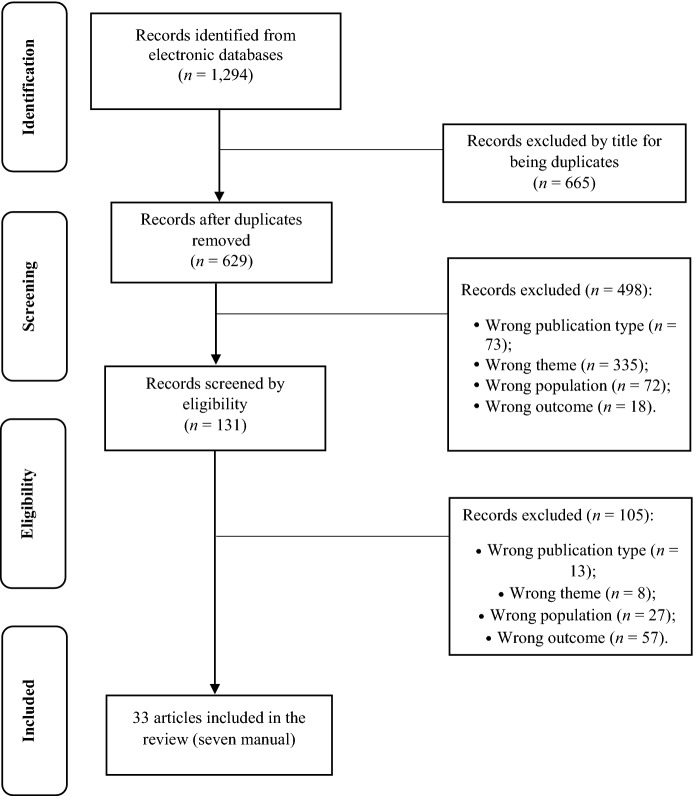
Flowchart of literature review process

Our database searches have retrieved 1,294 studies published between 2016 and 2023. After removing duplicates, the search outcome was reduced to to 629 unique studies. Afterwards, we examined the abstracts and excluded another 498 articles based on wrong publication type (*n* = 73), wrong theme (*n* = 335), wrong population (*n* = 72), or wrong outcome variable (*n* = 18). After full text analysis, 105 articles were eliminated, based the following criteria: wrong publication type (*n* = 13), wrong theme (*n* = 8), wrong population (*n* = 27), wrong outcome variable (*n* = 57) (Fig. 1). A total of 33 articles were included (seven from manual searching and 26 from the three databases). The objectives, sample (*N*, age, % male), and conclusions were extracted from each study.

## Results

Several studies have indicated different risk factors associated with gambling problems. At personal level, gender differences are clear and are mostly men the high-risk gamblers (Çakıcı et al., 2015; Çakıcı et al., 2021; Cunha et al., 2017; De Pasquale et al., 2018; Hing et al., 2016b; Hing & Russell, 2020; Volberg et al., 2017), young and single (Buth et al., 2017; Çakıcı et al., 2015; Çakıcı et al., 2021; Hing et al., 2016a; Hing et al., 2016b; Hing & Russell, 2020; Jiménez-Murcia et al., 2020; Volberg et al., 2017). These gamblers live alone and have been married less than 5 years (Çakıcı et al., 2021). In terms of education, they tend to be more educated (Buth et al., 2017; Çakıcı et al., 2015; Çakıcı et al., 2021; Hing et al., 2016a; Hing et al., 2016b; Hing & Russell, 2020; Jiménez-Murcia et al., 2020; Volberg et al., 2017), despite some studies relate to a low level of formal education (Buth et al., 2017; Cavalera et al., 2017; Cunha et al., 2017; Hing et al., 2016a; Volberg et al., 2017). In terms of occupation, studies found high-risk gamblers are working or studying full-time (Buth et al., 2017; Çakıcı et al., 2015; Çakıcı et al., 2021; Hing et al., 2016b; Hing & Russell, 2020; Jiménez-Murcia et al., 2020; Volberg et al., 2017), or unemployed (Hing et al., 2016a), have financial difficulties (Cowlishaw et al., 2016). At familiar level, usually grew up either in a single-parent home or with parents who had addiction issues (Buth et al., 2017; Cavalera et al., 2017). However, a study by Browne et al. (2019) sought to measure and assess 25 known risk factors for gambling-related harm. It concluded that sociodemographic risk factors did not demonstrate a direct role in the development of gambling harm, when other factors were controlled (Browne et al., 2019) (Table 1).

**Table 1:** Summary of Studies Characteristics

Study Identification	Objectives	Sample			Conclusions
		N	Age	% male	
Bellringer & Garrett (2021)	Aimed to extend current knowledge of gambling behaviors during COVID-19 restrictions by examining New Zealand data	*N* = 301	> 18	44	Behavioral risk factors included being a current low risk/moderate risk/problem gambler, a previously hazardous alcohol drinker or past participation in free-to-play gambling-type games
Bergamini et al. (2018)	To investigate the prevalence of at-risk gambling in a large, unselected sample of outpatients attending two community mental health centers, to estimate rates according to the main diagnosis, and to evaluate risk factors for gambling	*N* = 56	18–70 *M* = 48.7 *SD* = 13.7	48	Comorbidity with schizophrenia, bipolar disorder, unipolar depression and group B personality disorder
Bibby and Ross (2017)	To investigate the relationship between alexithymia and loss- chasing behavior in people at risk and not at risk for problem gambling	*N* = 58	*M* = 48.1 *SD* = 13.5	86	Alexithymia and problem gambling risk were significantly positively correlated
Biegun et al. (2019)	Validates one proposed measure of problem video gaming, in a Canadian undergraduate university student sample	*N* = 651	> 18	47	The video game problem is positively associated with average time spent playing the game, social alienation and online gaming motives such as competition, escape, coping, recreation and socialization In contrast, there is no correlation between problem gambling and several of its mental health correlates – depression, anxiety and stress
Black and Allen (2021)	Examined the association of baseline social, demographic, and clinical predictor variables with course in 48 older (≥ 60 years) and 57 younger (< 40 years) subjects with PPG in a prospective follow-up study	*N* = 105 (*n* = 57 younger;* n* = 48 older)	**Younger (< 40 years)** *M* = 27.5 *SD* = 8.0 **Older (≥ 60 years)** *M* = 66.6 *SD* = 6.9	**Younger** 81 **Older** 38	Predictors of disorderly gambling during follow-up included more severe PPG symptoms, more severe depressive symptoms, self-reported childhood neglect, gambling-related cognitive biases, and more role limitations due to physical health
Browne et al. (2019)	The present study attempted comprehensive measurement and evaluation of 25 known risk factors for gambling-related harm in order to determine which factors provided large and unique explanatory power	*N* = 1174	> 18 *M* = 43.4 *SD* = 15.3	40	Trait impulsivity is by far the most important risk factor. Overspending, less use of safe gambling practices, and more mistakes are the most significant proximal injury risks
Buth et al. (2017)	The aim of our study is to identify potential risk factors for disordered, problem, and at-risk gambling and to assess their respective relevance	*N* = 4,082 (*n* = 81 disordered; *n* = 72 problem; *n* = 121 at-risk; *n* = 2,808 social)	14 –65 **Disordered** *M* = 33.5 *SD* = 13.2 **Problem** *M* = 33.4 *SD* = 12.5 **At-risk** *M* = 40.4 *SD* = 12.5 **Social** *M* = 41.9 *SD* = 13.3	**Disordered** 79 **Problem** 68 **At-risk** 66 **Social** 54	Significant risk factors for gaming disorder include risk for alcohol use, poor mental health, young age (≤ 26 years), low formal education, growing up with a single parent, parents with addiction problems, and being working class Risk factors for problem gambling include parents with substance use problems, poor mental health and young age
Butler et al. (2019)	Examined the association between gambling problem severity and health risk behaviors, health, and wellbeing	*N* = 2,303	> 18	38	Low-severity players are approximately twice as likely to have poor mental health, and medium/high-severity players are three times more likely to have poor mental health than players with no issues
Çakici et al. (2021)	To investigate the prevalence and risk factors of PPG	*N* = 799	18 to 65	Lifetime non-PPG 86 Lifetime non-PPG 14	Being male, age range of 18–29, single, living alone and marry less than 5 years are the risk factors for PPG
Çakici et al. (2015)	To investigate the characteristics of adults’ participation in gambling, and to determine the prevalence of ‘problem and pathological gambling’ in North Cyprus	*N* = 966	18 to 65	–	Risk factors for becoming a problem and pathological gambler include being male, between the ages of 19–28, having a higher education, having a job, and being born in Cyprus
Cavalera et al. (2018)	Examined adult gambling behaviors from a local perspective in order to assess the adult at risk and problem gambler’s profile stratified by genre and by different forms of game	*N* = 4,773	18–94 *M* = 40.3 *SD* = 17.6	50	Both disadvantaged and problem gamblers are associated with men, and gamblers play more than one game and play strategy-based games
Cowlishaw et al. (2016)	Evaluated the prevalence and correlates of gambling problems in a U. S. representative sample reporting treatment for mood problems or anxiety	*N* = 3007	18–24	27	Lifelong gambling problems predicted relationship and financial difficulties, as well as marijuana use, but not alcohol use, mental or physical health, and use of health services
Cunha et al. (2017)	To identify characteristics with higher odds of distinguishing a group of PPG from (1) a group of gamblers without PG and 2) a SP	*N* = 331 (*n* = 162 NPG; *n* = 117 SP; *n* = 52 PPG)	> 18 **NPG** *M* = 33.6 *SD* = 10.9 **SP** *M* = 29.3 *SD* = 8.4 **PPG** *M* = 36.7 *SD* = 12.7	**NPG** 27 **SP** 44 **PPG** 83	The odds of being a PPG were higher for men with less education and less adaptive psychological relationship skills. Conversely, women with higher levels of education and more adapted psycho-relational functioning had higher odds of becoming NPs
De Pasquale et al. (2018)	To investigate the prevalence of Internet gaming disorder among Italian university students and to explore the associations between the former and dissociative phenomena	*N* = 221	18 to 25 *M* = 21.56 *SD* = 1.42	42	Data showed a positive correlation between Internet gaming disorder risk and some dissociative experiences: depersonalization and derealization, absorption and imaginative involvement, and passive influence
Delfabbro et al. (2012)	To conduct comparisons of the extent to which male and female problem gamblers report a range of potentially visible behavioral indicators of problematic gambling	*N* = 1,185 fortnightly gamblers (*n* = 338 problem gamblers as classified by the Problem Gambling Severity Index)	18–98 –		Men differed the most between problem gamblers and non-problem gamblers, either through signs of emotional distress or through trying to hide their presence on the gaming floor from others. Among women, signs of anger, declining caregiving and attempts to get credit were the most prominent indicators
Dennis et al. (2017)	To investigate whether unmet basic psychological needs evolve toward a level of psychological vulnerability that puts older adults who gamble at risk for becoming problem gamblers	*N* = 379	60–93 *M* = 68.0 *SD* = 6.9	42	Satisfaction of basic psychological needs also moderated the negative effects of socioeconomic status on risky gambling behavior
Dufour et al. (2019)	To examine factors influencing trajectories of poker players	*N* = 304	> 18 *M* = 32.5 *SD* = 11.5	88	Symptoms of depression were significant predictors of the third trajectory, while impulsivity predicted the second
Fluharty et al. (2022)	To examine a range of predictors of (i) gambling during the first strict lockdown, (ii) gambling more frequently during this strict lockdown compared to before lockdown, and (iii) continued increased frequency of gambling during the relaxation of restrictions	*N* = 556	> 18	51	As lockdown restrictions eased, individuals of ethnic minority backgrounds, who were current smokers, and with lower educational attainment were more likely to continue gambling more than usual
Flórez et al. (2016)	To examine the relation among these four factors in pathological gamblers	*N* = 144 (*n* = 44 PPG; *n* = 100 HC)	> 18 **PPG** *M* = 43.2 *SD* = 11.8 **HC** *M* = 50.3 *SD* = 8.4	**PPG** 98 **HC** 95	Pathological gamblers showed higher levels of impulsivity and more implicit attitudes towards gambling than the control group Active pathological gamblers showed more impulsivity, more explicit gambling cognitions and alcohol dependence than inactive gamblers
Gori et al. (2021)	To apply a Comprehensive Model of Addiction and to delve deeper the dimensions involved in the vulnerability and maintenance of the disease	*N* = 253	> 18 *M* = 47.8 *SD* = 12.4	83	Alexithymia may increase the risk of developing a gambling disorder, mediating the association between insecure attachment and dissociation
Hing and Russell (2020)	This study used an EGM-specific measure Problem Gambling Severity Index to achieve its aim of identifying risk factors specifically associated with problematic EGM play	*N* = 1,932	> 18 *M* = 41.84 *SD* = 16.46	53.1	High-risk EGM players tended to be younger, male, more educated, never married, had higher (although still modest) incomes, and were more likely to have problems with alcohol
Hing et al. (2016a)	To develop separate risk factor models for gambling problems for males and for females and identify gender-based similarities and differences	*N* = 8,917	18–24	–-	Important predictors of risk status among female gamblers included: age 18–24, not speaking English at home, living in a group household, unemployed or unemployed, private betting, EGM, scratch or bingo, and taking money from others Gambling is there for social reasons, to win money or for general entertainment Risk factors for men include: age 18–24, not speaking English at home, low education, living in a group household, unemployed or inactive, gambling on EGM, table games, racing, sports or lotteries, and winning at non-social gambling Reasons for money or general entertainment
Hing et al. (2016b)	To identify demographic, behavioral, and normative risk factors for gambling problems amongst sports bettors	*N* = 639	18–24	64	High-risk sports bettors were young, male, single, educated, full-time employed or full-time students
Hing et al. (2017)	Determine demographic, behavioral, and psychological risk factors for gambling problems on online EGMs, online sports betting and online race betting, and compare the characteristics of problematic online gamblers on each of these online forms	*N* = 162 (*n* = 64 non-problematic online EGM gamblers; *n* = 98 problematic online EGM gamblers)	> 18 **NPG** *M* = 39.6 *SD* = 15.3 **PPG** *M* = 36.8 *SD* = 12.7	**NPG** 69 **PPG** 71	Risk factors for online sports betting were being male, younger, lower income, born outside Australia, speaking a language other than English, more frequent sports betting, higher levels of psychological distress and negative attitudes towards gambling Risk factors for online match betting are being male, younger, speaking a language other than English, more frequent match betting, more forms of gambling, self-reported semi-professional/professional gamblers, illegal drug use while gambling, and negative attitudes towards gambling
Jiménez-Murcia et al. (2020)	To identify empirical clusters of GD based on several measures of the severity of gambling behavior and considering the potential role of patient sex as a moderator	*N* = 512	> 18 *M* = 43.0 *SD* = 13.5	92	The most severe GD traits were associated with single and multiple gambling preferences for non-strategic and strategic games, early gambling activity, higher impulsivity, higher dysfunctional scores for the harm avoidance and self-regulation personality traits, and more lifetime stressful life events
Kim et al. (2016)	Research had three aims; (a) explore gender differences (e.g., demographics, co-morbidities, gambling variables) among helpline callers using psychometrically robust measures, (b) assess whether gender predicts treatment utilization following contact and (c) assess whether systematic gender differences exist on gambling and psychosocial outcomes at 3-, 6- and 12-month follow-ups	*N* = 147	> 18 *M* = 39.3 *SD* = 13.4	43	Women compared to men described greater problem severity and shorter problem duration and were more likely to report video game machines as their most problematic form of gambling, greater distress, and lower quality of life Men, despite the lower severity and distress of the problem, were more likely to access treatment after contacting the helpline
Landreat et al. (2020)	To identify a typology of gamblers based on clinical and gambling characteristics, and to investigate factors associated with these different profiles in a large representative sample of gamblers	*N* = 628 (*n* = 256 NPG; *n* = 169 PGWT; *n* = 203 PGST)	> 18 *M* = 43.4 *SD* = 12.9	67	Anxiety or depressive symptoms may be the result of problem gambling problems
Medeiros et al. (2016)	To evaluate the association between anxiety symptoms, gambling activity, and neurocognition across the spectrum of gambling behavior	*N* = 143	18–29 *M* = 24.8 *SD* = 2.9	52	Anxiety may be associated with relevant clinical variables in the broad spectrum of gambling activity
Price et al. (2022)	Examined online gambling behavior during COVID-19 land-based gambling restrictions and associations with changes in mental health, impacts on household income due to the pandemic, financially focused motivations, and symptoms of gambling problems	*N* = 940	> 18 *M* = 33.9 *SD* = 16.4	53	An association between altered online gambling participation during COVID-19 and increased mental health problems, increased gambling problem severity, negative impact on household income, and a more financially oriented self-concept
Rodriguez-Monguio et al. (2017)	To assess the prevalence of co-occurring addictive behaviors and mental health disorders in pathological gamblers seeking treatment across all levels of care during a comprehensive period of time before casinos become operative in Massachusetts, in 2018	*N* = 869	> 18	71	The most common comorbidities in patients with a primary diagnosis of gambling addiction were anxiety disorders, mood disorders, and substance use disorders
Swanton et al. (2021)	To explore the relationship between financial well-being and changes in gambling behavior during the coronavirus 2019 (COVID-19) shutdown	*N* = 764	18–82 *M* = 43.8 *SD* = 14.8	85	Self-reported financial well-being has a strong negative association with gambling problems but is not related to gambling participation
Volberg et al. (2017)	To identify demographic characteristics, health-related behaviors, and gambling participation variables that statistically predicted the odds of being a problem or pathological gambler	*N* = 7,121	> 18 *M* = 27.8 *SD* = 9.1	42	Male participants, who smoked daily, suffered from depression, had increased effects of home gambling, had low levels of education, started gambling before age 21, or played in casinos, game rooms, or jogging tracks in the U.S. state. Problem or pathological gamblers were 1.5 to 2.3 times more likely last year than participants who did not fall into any of these categories
Wong et al. (2017)	To examine the main and interaction effects of gambling-related cognitions and psychological states on the gambling severity among a group of PPGs in Hong Kong	*N* = 177	18–65	100	Participants who reported a higher level of stress had more stable and serious gambling problems than those who reported a lower level of stress, regardless of their level of gambling-related cognitions

Physical and mental health are affected by gambling disorder (Black & Allen, 2021; Butler et al., 2019; Buth et al., 2017; Cowlishaw et al., 2016; Dennis et al., 2017), related to both psychiatric comorbidities (Bergamini, 2018), such as depression (Black & Allen, 2021; Dufour et al., 2019; Landreat et al., 2020; Rodriguez-Monguio et al., 2017; Volberg et al., 2017), anxiety (Landreat et al., 2020; Medeiros et al., 2016; Rodriguez-Monguio et al., 2017), and mood disorders (Rodriguez-Monguio et al., 2017), and substance use disorders (Bergamini, 2018; Cowlishaw et al., 2016; Rodriguez-Monguio et al., 2017; Wong et al., 2017), including excessive alcohol consumption (Browne et al., 2019; Hing & Russell, 2020). However, another study found that troublesome gambling and several of its mental health correlates—depression, anxiety, and stress—were not associated with troubling video game use (Biegun et al., 2020).

Regarding psychological risk factors, impulsivity was a significant risk factor (Browne et al., 2019; Dufour et al., 2019; Flórez et al., 2016; Gori et al., 2021; Jiménez-Murcia et al., 2020), demonstrating that active gamblers have more cognitive impulsivity and explicit gambling cognition than inactive gamblers. Also, Wong et al. (2017) found that negative psychological states (i.e., stress) significantly moderated the relationship between gambling cognitions and gambling severity. Participants who reported a higher level of stress had more stable and serious gambling problems than those who reported a lower level of stress, regardless of their level of gambling-related cognitions (Black & Allen, 2021; Jiménez-Murcia et al., 2020; Wong et al., 2017). Pathological gambling risk was positively correlated with dissociative experiences: depersonalization and derealization, absorption and imaginative involvement, and passive influence (De Pasquale et al., 2018). Also, alexithymia increases the risk of developing a gambling disorder (Bibby & Ross, 2017; Gori et al., 2021), and mediates the association between insecure attachment and dissociation (Gori et al., 2021). The results show a clear difference for the loss-chasing behavior (Bibby & Ross, 2017).

The analysis of gambling characteristics identified three distinct clinical traits of the gamblers: early and short-term onset (EOSC) (group 1), early and long-term onset (EOLC) (group 2), and late and short-term onset (LOSC) (Group 3) (Landreat et al., 2020). The incidence of gambling problems and the severity of gambling were higher in the EOSC group than in the other two groups. However, the onset age does not explain the gambling trajectories alone: the two clusters associated with the early onset age showed two distinct gambling trajectories, either a short-term evolution (~ 10 years) or a long-term evolution of the cluster. EOSC (~ 23 years) for the EOLC cluster. The EOLC cluster has a long history of gambling (35.4 years), they spend most money on gambling, with only 53.6% stopping gambling for at least a month. This cluster has a significantly higher preference for online gambling than other clusters. Although EOLC gamblers lived with their partners in most of the cases, they reported the lowest levels of family and social support related to gambling problems. An important feature was the absence of premorbid features of lifelong psychopathology before the onset of gambling problems. Most of LOSC gamblers preferred “pure” gambling (here understood as mere games of chance, as opposed to games that combine skill and chance). Women constituted the majority of the LOSC cluster, where game trajectories were the shortest observed in the study (Landreat et al., 2020).

Pathological gambling increases with the frequency (Cavalera et al., 2017; Hing et al., 2016b; Hing & Russell, 2020) and the diversity of the games (Cavalera et al., 2017; Jiménez-Murcia et al., 2020). Pathological gamblers engaged in a higher range of games of chance, and showed more impulsive responses towards gambling opportunities, including betting on live action games, individual bets, electronic gaming machines, scratch cards or bingo, table games, racing, sports or lotteries and winning non-social games (Hing et al., 2016a).

Furthermore, the main proximal predictors for high-risk gambling in electronic gaming machines (EGM) are higher desires, higher levels of misperceptions, higher session spend, longer sessions, separate EGM games, and EGM games in more locations (Hing & Russell, 2020). Normative influences from media advertising and significant others were also associated with a higher risk of problem gambling (Hing et al., 2016b).

A study that analyzed risk factors in online gaming concluded that more frequent gambling in online EGMs, substance use while gambling, and greater psychological distress were more frequent risk factors. Specifically, in an online sports betting group and an online racing betting group, researchers found that participants were mostly male, young, spoke a language other than English, were under greater psychological stress and showed more negative attitudes towards the game (Hing et al., 2017). However, sports betting gamblers had financial difficulties, while risk factors for online race betting gamblers included betting more often on races, engaging in more forms of gambling, self-reporting as a semi-professional/professional gambler, and used illicit drugs during the game (Hing et al., 2017).

Furthermore, moderate/highly severe gamblers were more likely to have a poor diet, engaged less in physical activities and had a poor general health than gamblers without problems. Also, tobacco use is associated with low and moderate/highly severe gambling. Low-severity gambling, opposing to moderate/highly severe gambling, was significantly associated with binge drinking and increased alcohol consumption. Unhealthy behaviors did tend to group together, and there was a scaled relationship between the severity of gambling problems and the likelihood of reporting at least two unhealthy behaviors. Compared to problem-free gamblers, low-severity gamblers were approximately twice as likely to have low mental well-being, and moderate/high-severity players were three times more likely to have low mental well-being (Butler et al., 2019).

To identify gambling trajectories in poker players, a latent class growth analysis was carried out over three years. Three gambling problem trajectories were identified, comprising a decreasing trajectory (1st: non-problematic-diminutive), a stable trajectory (2nd: low-risk-stable), and an increasing trajectory (3rd: problematic gamblers-increasing). The Internet as the main form of poker and the number of games played were associated with risk trajectories. Depression symptoms were significant predictors of the third trajectory, while impulsivity predicted the second trajectory. This study shows that the risk remains low over the years for most poker players. However, vulnerable poker players at the start of the study remain on a problematic growing trajectory (Dufour et al., 2019).

Regarding gender differentiation, studies have shown differences between the empirical groupings of men and women on different sociodemographic and clinical measures. In men, the number of DSM-5 criteria for disordered gambling (DG) reached the highest relative importance. This was followed by the degree of cognitive bias and the number of gambling activities. In women, the number of gambling activities reached the highest relative importance for grouping, followed by the number of DSM-5 criteria for PG. The relevance of the grouping was achieved by the cognitive bias (Jiménez-Murcia et al., 2020).

Women showed a preference for easy bets (easy bets are considered safer, therefore, with a greater chance of winning), electronic gambling machines, scratch cards or bingo for reasons other than socializing, earning money, or for general entertainment (Hing et al., 2016a). Women also reported greater problem severity and shorter problem duration, greater pain, and lower quality of life than men (Delfabbro et al., 2017; Kim et al., 2016). Men prefer to bet on EGMs, table games, races, sports, or lotteries and win non-social games (Hing et al., 2016a), and were more likely to exhibit aggressive behavior towards gaming equipment (Delfabbro et al., 2017). Men differed more between problem gamblers and non-problem gamblers, either through signs of emotional distress or trying to hide their presence in the game room from others. Among women, signs of anger, decreased care and attempts to obtain credit were the most prominent indicators (Delfabbro et al., 2017).

The risk of developing pathological gambling was higher for men with less education and less adaptive psychorelational skills. On the other hand, women with higher levels of education and more adapted psychorelational functioning were more likely to become pathological gamblers. Notwithstanding, the odds of being a pathological non-gambler (anything other than a pathological gambler) were higher for women with a high educational level and more adaptive psychorelational functioning (Cunha et al., 2017).

### Risk Factors for Increased Online Gambling During COVID-19

During 2020/21 almost one-quarter of online gamblers increased their gambling during lockdown (Bellringer & Garrett, 2021; Fluharty et al., 2022; Swanton et al., 2021), with this most likely to be on overseas gambling sites, instant scratch card gambling and Lotto (Bellringer & Garrett, 2021; Price et al., 2022). The sociodemographic risk factor for increased online gambling was higher education (Bellringer & Garrett, 2021), or low education (Fluharty et al., 2022), and financial difficulties related to COVID (Price et al., 2022; Swanton et al., 2021).

The studies indicate a link between change in online gambling involvement during COVID-19 and increased mental health problems (Price et al., 2022), including stress from boredom (Fluharty et al., 2022), and higher levels of depression and anxiety (Fluharty et al., 2022; Price et al., 2022).

Behavioral risk factors included being a current low risk/moderate risk/problem gambler, a previously hazardous alcohol drinker (i.e., excessive) or past participation in free-to-play gambling-type games (Bellringer & Garrett, 2021), and alcohol consumption (Fluharty et al., 2022; Swanton et al., 2021). Financial well-being showed strong negative associations with problem gambling and psychological distress (Swanton et al., 2021).

As lockdown restrictions eased, ethnic minority individuals who were current smokers and were less educated were more likely to continue gambling more than usual (Fluharty et al., 2022).

## Discussion

With this systematic review, we aimed at exploring what are the risk factors for the development/maintenance of gambling disorder. We also searched the literature for information on differences between pre-pandemic gambling patterns and gambling patterns today. A total of 33 studies examined risk factors associated with gambling problems in adults.

Studies, with mixed samples, have shown several risk factors associated with risk problems for problem or pathological gamblers, namely being male, young, single or married less than 5 years, living alone, having a low level of education, and having financial difficulties.

As for relationships, pathological gamblers have greater difficulties in family and social relationships than non-players (Cowlishaw et al., 2016; Landreat et al., 2020). And they even increase the risk of gambling when they grew up with a single parent (Buth et al., 2017) or parents with addiction problems (Buth et al., 2017; Cavalera et al., 2017; Hing et al., 2017).

About health, there is a consensus that gambling addiction decreases quality of life, a reflection of worse mental health (Buth et al., 2017; Butler et al., 2019; Cowlishaw et al., 2016; Dennis et al., 2017; Delfabbro et al., 2017). Studies have shown a comorbidity of gambling problems with higher levels of stress (Hing et al., 2017; Wong et al., 2017), higher levels of impulsivity (Browne et al., 2019; Dufour et al., 2019; Gori et al., 2021; Jiménez-Murcia et al., 2020; Flórez et al., 2016), cognitive distortions (Black & Allen, 2021; De Pasquale et al., 2018), and various pathologies, namely, anxiety (Fluharty et al., 2022; Landreat et al., 2020; Medeiros et al., 2016; Rodriguez-Monguio et al., 2017), schizophrenia (Bergamini, 2018), bipolar disorder (Bergamini, 2018), depression (Bergamini, 2018; Black & Allen, 2021; Dufour et al., 2019; Fluharty et al., 2022; Landreat et al., 2020), alexithymia (Bibby & Ross, 2017; Gori et al., 2021), mood disorders (Rodriguez-Monguio et al., 2017), and substance use disorders (Bergamini, 2018; Buth et al., 2017; Butler et al., 2019; Browne et al., 2019; Cowlishaw et al., 2016; Flórez et al., 2016; Fluharty et al., 2022; Hing & Russell, 2020; Hing et al., 2017; Rodriguez-Monguio et al., 2017).

As for the type of game, gamblers who played more than one game, and had longer gambling sessions, were at greater risk of problem gambling (Cavalera et al., 2017; Hing et al., 2016a; Hing & Russell, 2020; Jiménez-Murcia et al., 2020).

However, two studies presented different data (Biegun et al., 2020; Çakıcı et al., 2015). Biegun et al. (2020), did not find an association between problem gambling and various mental health correlates, such as depression, anxiety, and stress. In another study, players had higher levels of education and were employed, contrary to data found so far. However, it is necessary to bear in mind that the study was developed in Cyprus and, as the authors themselves mention, it is a country with sociocultural characteristics, such as a history of colonization, socioeconomic problems, and high unemployment (Çakıcı et al., 2015), which may justify that only people with income can become addicted to gambling.

With the COVID-19 pandemic, online gamblers have increased their gambling (Bellringer & Garrett, 2021), aggravating the psychological and social consequences for people with problematic gambling behaviors (Håkansson et al., 2020; Yayha & Khawaja, 2020). The authors highlighted the removal of protective factors, including structured daily life (Yayha & Khawaja, 2020), boredom (Fluharty et al., 2022; Lindner et al., 2020), depression and anxiety (Fluharty et al., 2022), as well a financial deprivation (Price, 2020; Swanton et al., 2021), as the main reasons for the increase in gambling problems during the COVID-19 pandemic. It is well known that the daily lives of many people have been substantially altered, with a high degree of homeschooling for school children and students (Tejedor et al., 2021), also with likely negative effects for young people and their families (Thorell et al., 2021). Likewise, restrictions related to COVID-19 and changes in the lives of many people have led to significant job insecurity, unemployment, and financial problems, as well as fear of illness and mortality, which has increased emotional distress (Shakil et al., 2021; Swanton et al., 2021). Researchers have expressed concerns that COVID-19 would have consequences for the mental health (Holmes et al., 2020; Zheng et al., 2021), as well as substance use disorders, and it is important to adapt treatment during the pandemic. (Marsden et al., 2020). The increase in the incidence and prevalence of behavioral addictions and the relevance of the early onset of the problem of gambling disorder, with its serious consequences, make it necessary to better understand these problems to develop and adapt prevention and treatment programs to the specific needs of according to sex and age. Furthermore, understanding gender-related differences is of great importance in treating behavioral addictions.

The growing availability of gambling in recent decades, a low social knowledge about gambling disorders, and a perception of gambling more in terms of moral weakness than a psychological/psychiatric disorder have an impact on the social acceptance of gambling behaviors (e.g., Hing et al., 2015; Petry & Blanco, 2013; St-Pierre et al., 2014).

This systematic review presents limitations. As in all systematic reviews, there is the risk of reporting bias. As only studies published in identifiable sources were included, unpublished studies may be more likely to not have significant results, thus indicating the absence of risk factors in the involvement in problematic or pathological gambling that we have analyzed. For this reason, we had no constraints regarding geographic and linguistic criteria. Also, the adherence to the PRISMA guidelines, including definition of accurate inclusion and exclusion criteria, the use of independent reviewers, as well as the efforts to diminish publication bias, strengthen this systematic review and better elucidate about risk factors in the involvement in problematic or pathological gambling. Another limitation of this study is the little literature on the post-COVID pathological gambling, which does not allow us to draw conclusions from comparisons. Future research would benefit from making comparisons, not just across gender, but also across culture. Researchers should further explore and understand how cultural environments influence the development of problematic gambling.

Treatment providers must consider the specificities of people with gambling disorders. Therefore, a strong educational/training background for therapists and other professionals, considering the problem of gambling disorders in the diagnosis, a better adaptation of the contents of therapeutic programs, and the creation of materials used in therapy adapted to the patient’s needs, would be very much advisable. It would also be helpful to establish therapeutic groups, ideally with at least a couple of patients with gambling disorders.
